# Toward a Socio-Territorial Approach to Health: Health Equity in West Africa

**DOI:** 10.3390/ijerph14010106

**Published:** 2017-01-22

**Authors:** Lucie Vialard, Clara Squiban, Florence Fournet, Gérard Salem, Ellen E. Foley

**Affiliations:** 1 Laboratoire Dynamiques Sociales et Recomposition des Espaces (LADYSS), Université Paris Ouest Nanterre, Nanterre 92000, France; lucie.vialard@gmail.com (L.V.); clara.squiban@gmail.com (C.S.); salem.gerard@gmail.com (G.S.); 2 Unité Mixte de Recherche Maladies Infectieuses et Vecteurs: Ecologie, Génétique, Evolution et Contrôle (MIVEGEC), Institut de Recherche pour le Développement, Montpellier 34394, France; florence.fournet@ird.fr; 3 Centre Population et Développement (CEPED), Université Paris Descartes-Institut de Recherche pour le Développement, Paris 75006, France; 4 International Development and Social Change, IDCE, Clark University, Worcester, MA 01610, USA

**Keywords:** socio-territorial approach, health disparities, hypertension, diabetes, urban health, West Africa

## Abstract

This study contributes to the literature about the effects of space and place on health by introducing a socio-territorial approach to urban health disparities in West Africa. It explores how urban spaces, specifically neighbourhoods, are shaped by social and economic relations and strategies of territorial control. We examine the potential influence of socio-territorial processes on vulnerability to disease, access to medical care, healthscapes, and illness experiences. Our research was conducted in Senegal and relied on a mixed methods design. We identified four neighbourhoods that represent the socio-spatial heterogeneity of the city of Saint-Louis and utilized the following methods: geographic and anthropological field research, household surveys, health knowledge and behaviour surveys, clinical exams, and illness interviews. Our results highlight the socio-territorial processes at work in each neighbourhood, clinical findings on three health measures (overweight, high blood pressure, and hyperglycaemia) and health experiences of individuals with hypertension or type II diabetes. We found significant differences in the prevalence of the three health measures in the study sites, while experiences managing hypertension and diabetes were similar. We conclude that a socio-territorial approach offers insight into the complex constellation of forces that produce health disparities in urban settings.

## 1. Introduction

Over the past two decades there has been an explosion of interest in understanding the complex effects of space and place on variations in health outcomes [1]. The growing popularity of mapping tools and Geographic Information Science has pushed more and more social scientists to “make a place for space” and spatial thinking in their research design and analysis [2]. A number of sophisticated debates continue among geographers, sociologists, anthropologists, and others whose research attempts to explain how urban places shape epidemiology and experiences of health and disease [3,4]. Following Paul Farmer, if disease is inequality written on the body, in what ways do places, particularly urban places, also become embodied and evident in health in/equities (e.g., differences in health in which marginalized social groups experience worse health or greater vulnerability to morbidity and mortality than more socially advantaged groups)?

In this article, we intervene in these debates by arguing for an approach that we call the socio-territorial production of health. Much of the existing literature attributes spatial variations in health outcomes to either compositional factors (the social and material circumstances of people living in a particular place) or to the contextual factors of the place they live (social, material, environmental or political characteristics) [5]. Building upon the “relational approach” in health research advocated by Steven Cummins and colleagues [6], we suggest that compositional and contextual factors are mutually constitutive. In other words, urban places are produced by the social relations of their residents, and the social organization and health status of residents also reflects the characteristics of the particular places where they live. We suggest that the key to understanding health disparities within given urban contexts lies in examining the historical and contemporary processes through which these places are produced and managed politically, socially, and economically. To arrive at the origins of health disparities, we call for examining how the variation in health status of different neighbourhoods is produced by unique histories of urbanization and contemporary use and management of urban space.

A socio-territorial approach allows us to interrogate the relationships among urban populations, urban spaces, and health, and provides a framework for understanding how societies manage space and how space in turn influences society and social organization [7]. The concept of territory is rarely employed in the Anglophone health geography literature. In political geography, this term conveys political power in an administrative sense [8]. Most authors prefer to use the concept of place to indicate the social, cultural and historical meanings of a localized site. Our approach assumes that space is shaped by both social and territorial control strategies. We aim to examine the ways that policies and politics take material form in a specific space and how this materiality of politics may influence health inequalities [9]. We assume that health risks and disparities do not result simply from a given physical environment. They stem from a constellation of environmental and social forces and they are the cumulative result of the social and political management of particular places over time.

How do people stay well in the city? Once sick, how do they get well? Health-seeking behaviour in urban areas takes place amidst a tremendous diversity of medical practitioners, techniques, and therapies. Environmental exposures and risks, health needs, and access to treatment are all mediated by class, gender, education, and household structure. While much existing research on medical pluralism explores the influence of therapy-managing groups and cultural norms on therapeutic itineraries [10,11], our approach extends this inquiry to examine the potential influence of place on health behaviour, access to care, and therapeutic strategies [7].

Health inequalities have often been thought of as strictly social [12] or strictly spatial with spatial patterns typically being seen as the geographic expression of social differences [13,14]. More recent research suggests that space is not just the simple expression of spatialized social inequalities, but a variable that may partly explain these disparities. This insight requires that we move beyond individualized notions of risk to consider the notion of unequal or risky urban spaces [7,15,16,17].

Our understanding of socio-territorial constructions of health goes beyond measuring socio-spatial health disparities and place effect, to how the ongoing production of the city generates health and vice versa. More specifically, strategies of social and territorial control can lead to the spatial segregation of marginal and powerless populations and differential access to health-enabling resources. Vulnerable populations are often clustered together in marginal places and they typically do not have the same access to and mastery over urban resources as other citizens. The way people move in the city (or are restricted from moving) is a reflection of power relations [18]. These urban processes produce inequalities in exposures, unmet health needs and uneven access to medical care. On the other hand, lack of health providers in a specific neighbourhood may motivate stakeholders to initiate their own health projects.

To develop this approach, the SANTINELLES project selected two West African mid-sized cities, Saint-Louis, Senegal, and Bobo Dioulasso, Burkina Faso, to examine the potential influence of diverse socio-territorial processes on population health and health inequalities. Like many West African cities, Saint-Louis and Bobo Dioulasso were each part of the French colonial empire until the early 1960s; thus, they were subject to segregationist urban policies, followed by post-Independence clientelist politics, and neoliberal economic policies, including structural adjustment [10,19,20,21,22,23]. We assume that this historical legacy, subsequent modes of economic and social organization and the urban environment contributes to intra-urban variations and creates differential health risks among neighbourhoods.

In this article, we present data only from Saint-Louis, Senegal. First, we demonstrate the variety of socio-territorial processes at work in urban settings. We then highlight how these socio-territorial processes may influence health inequalities. The objective of the study was to examine how varying socio-territorial processes influenced vulnerability to a number of different health conditions. In this article, we focus on disparities in being overweight, in arterial hypertension and in type II diabetes to illustrate how socio-territorial processes at the neighbourhood level produce different levels of vulnerability to these conditions.

## 2. Methods

**Figure 1:**
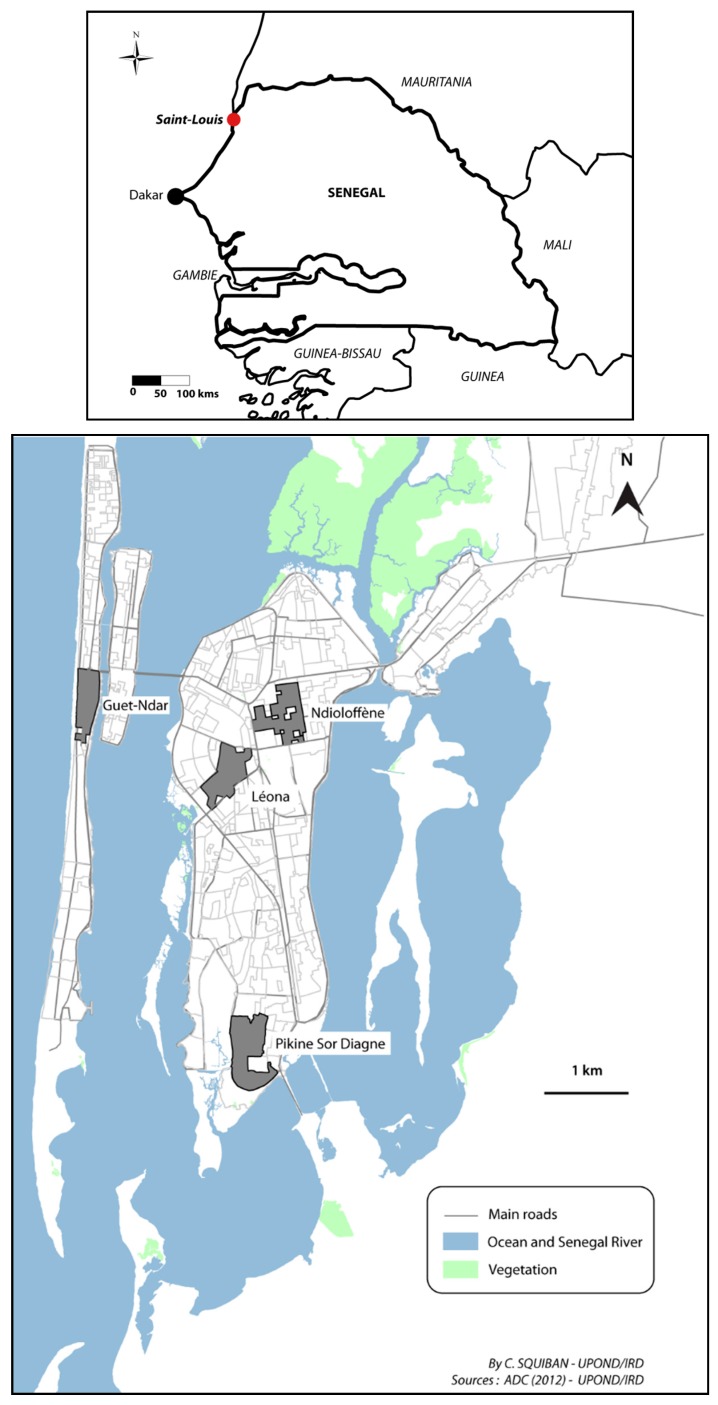
Location of the study areas in Saint-Louis.

Our study of Saint-Louis, Senegal had several objectives: to determine the socio-territorial processes at work in different sites, to uncover the genesis of urban health inequalities and to measure these inequalities. We selected four neighbourhoods with the aim of representing the heterogeneity of urban spaces and urban dwellers in the city. As the larger study aimed at comparing socio-spatial processes of health in two mid-sized West African cities, we chose standard and available indicators to permit this comparison. The neighbourhoods varied along lines of date of origin, population densities and built environment densities, presence of schools, infrastructure such as electricity and running water, and physical proximity to health care structures. The four sites selected in Saint-Louis were Guet Ndar, Ndioloffène, Léona, and Pikine Sor Diagne (Figure 1).

The research design included three overlapping phases: phase 1 included qualitative field work on socio-territorial processes in each neighbourhood (January 2013 to June 2014); phase 2 included a household survey conducted in tandem with clinical exams and a socio-medical questionnaire (August–September 2014); and phase 3 comprised 80 qualitative illness experience interviews conducted over a six-month period in 2014 (June–December 2014). Funding was provided by the Agence Nationale de la Recherche (ANR12-INEG-007) and the Centre National de la Recherche Scientifique (PEPS107-IE) provided methodological support. This study was approved by the Comité National d’Ethique pour la Recherche en Santé (CNERS) of Senegal (authorization No. SEN14/29).

### 2.1. Analysis of Socio-Spatial Contexts

**Figure 2:**
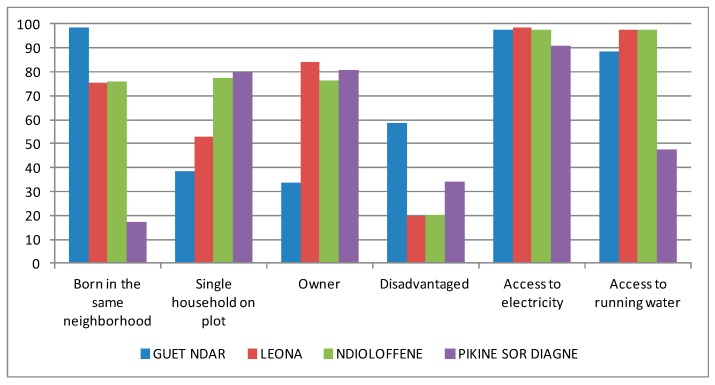
Main characteristics of the surveyed households.

Our research design relied on a random spatial sampling of 1003 households in the four selected neighbourhoods (272 in Guet Ndar, 241 in Léona, 256 in Ndioloffène, 234 in Pikine Sor Diagne). In each household, we interviewed the household head to collect data about length of residence in the neighbourhood, home ownership (owner or renter), the presence of one or more households in the same plot, socio-economic status (based on ownership of a variety of consumer goods), access to electricity, and access to running water (Figure 2). Significant differences between the neighbourhoods could be observed with all the variables (*p* < 0.01) except for the number of households in a same plot and for access to electricity.

To complement the household survey data, we conducted semi-structured interviews (*n* = 44) with key informants in each neighbourhood (elected officials, neighbourhood residents, health committee members, medical personnel, youth and sports association members, women’s group members) to document the history of each neighbourhood, the means of control over public and private spaces, the religious and political landscape, how well neighbourhood health facilities were working, the presence of medical actors and health interventions, the existence and funding of development initiatives, and political and economic tensions and processes. Interviews were conducted in French and lasted 30 to 75 min. Interviews were recorded and then transcribed. The main themes of our analysis were the genesis of contemporary socio-spatial patterns, relationships among neighbourhood-based and municipal actors, and local development dynamics.

### 2.2. Health Status, Health Behaviour and Health Inequalities

In each neighbourhood, we collected data to assess health status, knowledge of key health conditions, and health behaviour. In the course of the household surveys, we invited one adult (age 35–59 years) chosen at random from among the household members to participate in follow-up clinical exams and a socio-medical survey. The adult age range corresponds with adults of working age as well as the likely age of onset of the non-communicable diseases of interest: hypertension and type II diabetes. Those who agreed to participate came to a designated site where they received medical exams and were interviewed about their health knowledge and health practices.

Adults were interviewed about age, sex, education level (not educated), place of birth, well-being in the neighbourhood (wish to stay), and perception of health status (poor or not) (see Table 1).

Clinical exams included taking vitals (height and weight), blood pressure readings, and fingerprick to test blood glucose levels. The findings presented here include overweight, blood pressure, and hyperglycaemia. Overweight corresponds to a BMI > 25; high blood pressure is considered to be equal or higher than 140 or 90 mmHg or if the person is under treatment; people were considered at risk for hyperglycaemia when their fasting blood sugar was ≥110 mg/dL). We used Chi square tests to measure statistical correlation between contributing factors, overweight and high blood pressure. For hyperglycaemia, given the low number of diagnosed cases, we used the Fisher’s exact test. In case of risk of hypertension or diabetes, people were asked to visit a physician and the medical visit was paid for by the project (initial consultation, exams and treatment for one month). Of a target goal of 1000 adult participants (250 from each neighbourhood), our sample included 214 persons from Guet Ndar, 224 from Ndioloffène, 186 from Léona and 191 from Pikine Sor Diagne.

**Figure 3:**
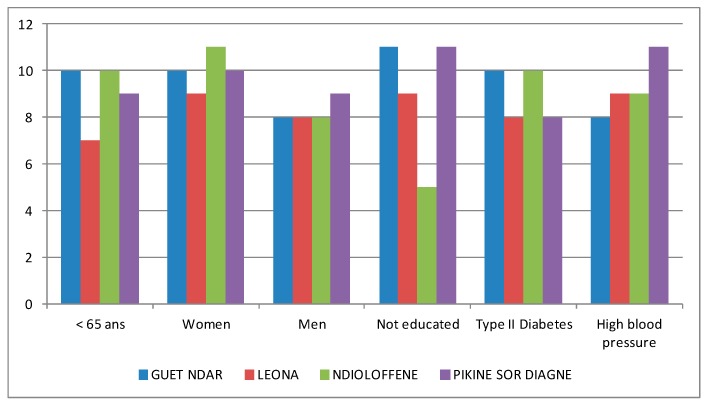
Characteristics of illness interview participants.

To understand the challenges facing individuals living with hypertension and type II diabetes, we used a snowball sampling method to identify men and women in each neighbourhood who had been diagnosed with arterial hypertension (AH) or diabetes at least two years prior to the time of the study who were then invited to participate in illness interviews. Given the challenges of identifying interview participants, we did not restrict the age of interviewees to adults 35–59. We conducted 73 interviews total, 36 with individuals with type II diabetes and 37 with individuals with hypertension. See Figure 3 for an overview of the characteristics of interview participants.

We conducted in-depth interviews with these patients to document their experiences with these conditions. Interviews were conducted in French and Wolof. The interviews were designed to elicit each person’s thoughts about health issues in their neighbourhood, their experiences with diagnosis and management of AH and diabetes, how these conditions had affected household and family dynamics, the cost and accessibility of medical care, use of non-biomedical therapies, and their long-term aspirations for health and well-being. Interviews were simultaneously translated and transcribed into French, and were then analysed using the qualitative software program MAXQDA.

## 3. Results

### 3.1. Socio-Territorial Processes in Saint-Louis

Here we highlight the distinct profiles of the four selected urban sites. Their histories, recent patterns of urbanization, and social, economic, and political arenas demonstrate the heterogeneity that characterizes mid-sized cities in West Africa.

Saint-Louis was created as a colonial trading fort in 1659. It quickly became an economic crossroads and served as the capital of French West Africa until 1902. After serving as the capital of Senegal and Mauritania, it remained a major urban colonial hub until independence in 1960. During this period, the four neighbourhoods we selected for our study developed varying relationships with the colonial administration.

#### 3.1.1. Guet Ndar, from Fishing Village to Economic Hub of Saint-Louis

Guet Ndar, located on the Langue de Barbarie between the Atlantic Ocean and the Senegal River, is the oldest district of the city. It was originally a Wolof fishing village and fishing-related activities remain the major source of income. The village existed prior to colonial rule and its residents resisted the imposition of French law. Once complete resistance to the colonial authorities was no longer possible, residents made strategic compromises with the French administration that guaranteed them greater autonomy and jurisdiction over neighbourhood affairs than most other colonial subjects [19,24]. This historical legacy is evident in the present. Guet Ndar remains a distinct neighbourhood with a clear social, economic, and political boundary separating it from the rest of the city.

**Figure 4:**
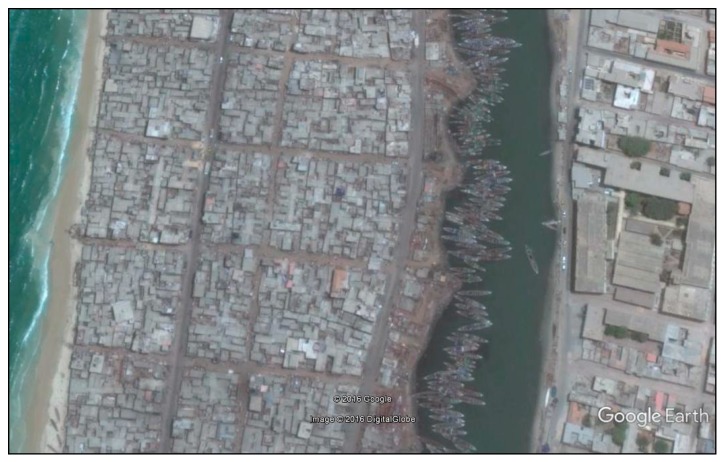
Aerial view of Guet Ndar along the Atlantic Ocean, showing the high building density in contrast to the Island district on the other side of the river (Google Earth^®^).

Guet Ndar, among other unique characteristics, is renowned for being one of the most densely populated and densely built districts in West Africa. The significantly higher number of households in each plot is a revealing sign of this density (see Figure 4). Several attempts to alleviate the highly congested neighbourhood by moving Guet Ndarians to housing projects elsewhere in the city have failed. At the time of writing, youth in Guet Ndar were in violent conflict with city police after the demolition of neighbourhood buildings as part of an urban planning project, “Projet Assainissement Concerté Total Intégré du quartier Guet Ndar.” Their livelihood as fishermen requires proximity to their fishing boats and the sea, and their place of residence is inseparable from their collective identity. They simply cannot be Guet Ndarian any place else. According to our survey, almost all residents were born and have always lived in the neighbourhood (98.5% vs. 17.1% in Pikine Sor Diagne for example, Figure 2). As a result, ever-expanding families occupy a very small strip of land between the Senegal River and the Atlantic.

There are several ways that the high population densities in Guet Ndar materialize in the urban landscape. Many buildings appear to be dilapidated and in various states of decay. The number of adults and children in each concession means that there is inadequate space for the numerous tasks of production and reproduction. A casual tour of Guet Ndar reveals that many domestic tasks, including meal preparation and laundry, take place in open areas and lanes between tightly packed houses. These domestic tasks compete for space with other individuals and activities: animal husbandry (largely sheep and goats), children seeking space for recreation, and other neighbourhood economic activities including the repair of fishing nets and pirogue engines. The physical appearance of the neighbourhood gives the impression of collective economic deprivation and urban disorder.

Similarly, according to the socio-economic index that we developed based on material wealth, almost 58% of the Guet Ndarian households surveyed were classified as being economically disadvantaged. Despite these findings, tremendous amounts of capital circulate through Guet Ndar, and the fishing economy, though increasingly threatened by overfishing and the allotment of concessions to other countries, is still one of the strongest economic sectors in the city. Contrary to the obvious lack of investment in home maintenance and the high number of people living in tight quarters, Guet Ndar has long been the major economic hub of Saint-Louis [19,25,26]. Prior research in Guet Ndar has found a strong preference for consumption of fishing profits or their reinvestment in fishing equipment, rather than investment in property (possibly due to precarious ownership and unclear lines of inheritance) [25,26]. If the landscape appears dilapidated, it is the result of a unique social history and urban political ecology rather than poverty.

Given the economic focus on fishing in Guet Ndar to the near exclusion of all other livelihood strategies, affluence does not necessarily coincide with levels of schooling. According to our survey, around 70% of Guet Ndar residents have never attended school. In line with the fishing economy, children are often recruited at early ages to work on the family boat, though in recent years more children are attending school for longer periods of time. The family unit is the basic unit of production and children are of great economic value to their families. The dependence of Guet Ndarians on fishing may as yet prove to be untenable in the future. Young men from Guet Ndar, concerned about overfishing, the increasing costs of boats and motors, and their long-term prospects as fisherman, have been at the epicentre of clandestine migration to Europe [26]. It may be that Guet Ndar is on the cusp of significant socio-economic shifts.

Regarding access to medical care, a public health post is available for residents in the southern part of Guet Ndar and is mainly visited by women and children (see Figure 2). Residents from the northern part tend to seek care at the health post of “Ndar Toute,” the neighbouring district to the north. The regional hospital is located in very close proximity on the Island, on the other side of the bridge. Despite its population density, housing shortage, and congested and waste-strewn public spaces, Guet Ndar has a relative abundance of medical facilities in close proximity compared with the other neighbourhood sites in Saint-Louis.

#### 3.1.2. Ndioloffène and Léona: Two Colonial Neighbourhoods, Two Distinct Histories

In contrast to the pre-colonial fishing village of Guet Ndar, Ndioloffène and Léona were the product of urban sprawl during the colonial period [19]. Increasing population density on the Island and the Langue de Barbarie led residents to emigrate to the continent. Ndioloffène developed during the 1830s as a result of this urban expansion, and it also received urban migrants from other parts of the continent, including from the Djolof region of Senegal (hence the neighbourhood name) and from Mali at the end of the 19th century. Historically, Ndioloffène was a mixed neighbourhood whose residents included colonial officials, African civil servants, and mixed-race (métis) families.

Léona was originally an uninhabitable part of Ndioloffène due to flooding and wetlands. In the 1930s, the city government filled in Léona’s marshland with sand mined largely in Pikine Sor Diagne (not yet a municipal district at this time). Many dwellings, including public housing, were built in this district to accommodate new residents who came from all corners of the city and beyond to work as civil servants in the French colonial administration.

**Figure 5:**
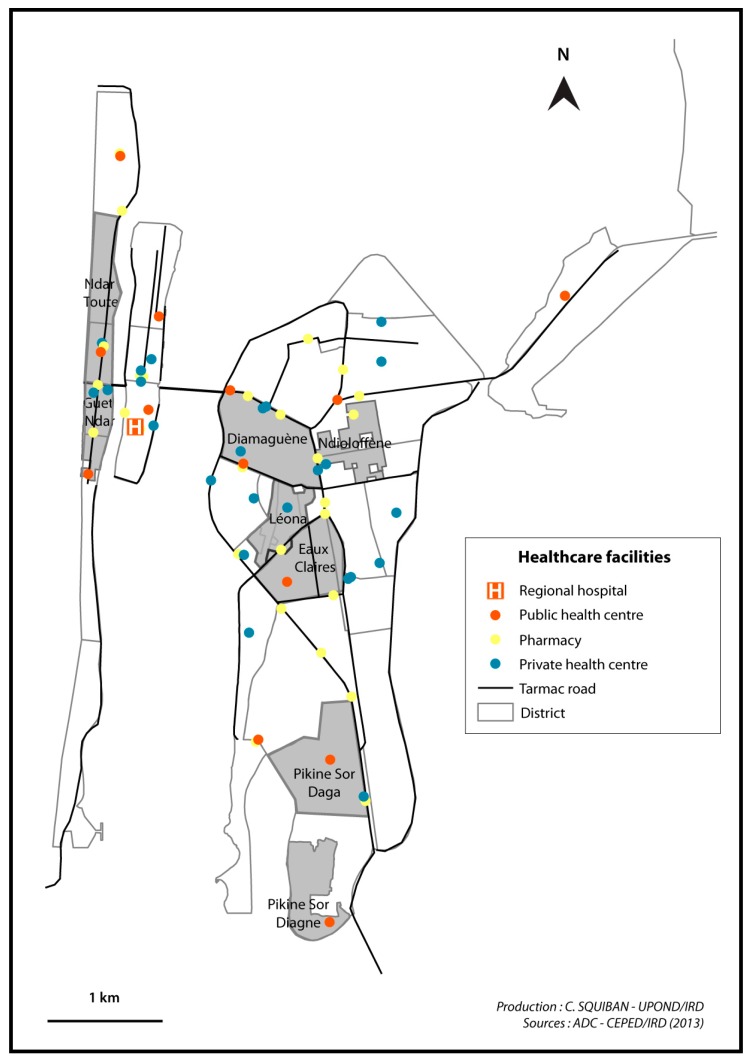
Location of the health care facilities in Saint-Louis (2014).

These two districts were both subject to colonial urban planning and are presently characterized by large familial homesteads, wide streets and good access to various public infrastructures (running water and electricity). Although neither Ndioloffène nor Léona has a public health post, access to medical care does not pose a geographic problem for neighbourhood residents: their neighbouring districts (Diamaguène and Eaux Claires) each have a public health post in which residents seek care. In addition, a variety of private medical facilities are available (see Figure 5).

Léona and Ndioloffène have the most highly educated residents among the four study sites in Saint-Louis. The first settlers of Ndioloffène, who came from rural regions, interacted with the French colonial population and generally sent their children to school. Most current residents went at least to primary school (70% to 80% according to our survey) and these neighbourhoods are considered the intellectual hub of Saint-Louis. However, slight differences can be observed regarding their social composition. According to the socio-economic index, the share of affluent households is higher in Ndioloffène than in Léona (50.8% vs. 41.9% respectively), whereas Léona has a larger middle class compared to Ndioloffène (38.6% vs. 28.9% respectively) (see Table 2). Numerous famous elected officials and political figures live in Ndioloffène, whereas Léona counts among its residents civil servants and middle-class merchants.

#### 3.1.3. Pikine Sor Diagne, the Face of Urban Sprawl

Pikine Sor Diagne, now a peripheral district of Saint-Louis, was a remote, rural village of farmers and fishermen for most of the 19th and 20th centuries. It was largely isolated from and irrelevant to the colonial administration, whose headquarters were on the island of Saint-Louis (next to Guet Ndar). In the 1970s, the Senegalese state annexed agricultural lands at the city’s periphery to address urban congestion. Nevertheless, the original population of Pikine Sor Diagne was granted titles as owners of their property. Pikine Sor Diagne officially became part of the Saint-Louis municipal district in 1982.

Due to this late integration into the city and lack of formal urban planning, most of the neighbourhood has no household access to water or sanitation services. Most residents rely on public water taps or itinerant water sellers. As for health care access, there is a small dispensary available for the neighbourhood that offers some essential medicines and first aid (see Figure 5). According to our interviews, residents tend to consult at the health post of Pikine Sor Daga which is about two kilometres away.

Today, Pikine Sor Diagne remains spatially landlocked: the lack of public infrastructure and the low population and built densities give the impression of an unplanned area. In actuality, the neighbourhood will become part of an ambitious urban development project led by the state which has not started yet in this part of Pikine. This situation led to land speculation: in recent years, the earliest neighbourhood residents sold their lands at a low price to newer residents who were attracted by this opportunity. The relative small number of residents who were born and still live in the neighbourhood is illustrative of this phenomenon (see Figure 2 and Table 1). Today, although Pikine Sor Diagne is still sparsely built, there are no remaining plots for sale. The neighbourhood is currently undergoing significant changes, especially regarding the social composition of the population: according to our socio-economic index, the households surveyed are equally distributed between the three categories of socio-economic status (disadvantaged/middle class/upper class). This social diversity is also illustrated by their education levels: around 50% of residents have never attended school. Social reconfiguration is also tangible in the urban landscape as mud brick houses now coexist with luxurious hostels. Many residents desire to stay in the neighbourhood, probably in view of the planned improvements to the neighbourhood.

These particular historical legacies leave their imprint on neighbourhood landscapes. Each of these neighbourhoods reflects a unique confluence of social, economic, political, and environmental factors that have shaped the social identities, social cohesion, and potentially the health of their inhabitants. The city of Saint-Louis is not one place, but a constellation of highly distinct places.

#### 3.1.4. How Politics Materialize in Physical Space: The Example of Local Development

Since the 1990s, the municipality of Saint-Louis has implemented a decentralized model for local development at the neighbourhood scale. As a consequence, each neighbourhood was required to create local councils in order to manage local development projects under the authority of the municipality. However, this model was not taken up equally by the various locally based actors, which has led to inequalities in local development [27]. In this section, we offer two relevant examples illustrating these distinguishing processes in Léona and Pikine Sor Diagne.

In Léona, as detailed previously, the majority of inhabitants are well-educated and several of them have gained expertise in project management. Social cohesion seems to be relatively strong as local actors work together for a variety of ends. The powerbrokers have proven to be masterful at securing external funding for local development projects from a variety of international donors. At the time of our research, Léona was the recipient of significant development assistance: a German association supported the creation of a social centre for uneducated youth; another NGO based in Luxembourg funded a kitchen in which to teach food service in the same centre; and two different Spanish associations financed a prevention campaign and the renovation of the local health centre. In short, the locally elected neighbourhood council has become proficient in financial solicitation.

This ability of Léona’s elected officials to raise external funds proved unpopular with city government, which attempted to collect a portion of Léona’s development financing. To circumvent the ability of the municipality to collect its share of the revenue, elected leaders in Léona created an independent association (comprised of exactly the same representatives as those who serve on the neighbourhood council) that operates outside of the elected neighbourhood councils that are part of the municipal government. This committee now operates independently and free of the municipality’s oversight. Thanks to this tremendous mobilization, Léona has a great degree of financial and political autonomy from the municipality. It can initiate project ideas, solicit donors, and implement projects free of the municipality’s oversight.

While Léona benefits from health promotion projects due to this exceptional ability to fundraise for local development projects, other districts are far from attracting such resources. As an example, the only health project conducted at the time of the research in Pikine Sor Diagne and funded by external financing was the building of a small dispensary. In this neighbourhood, the few local development projects generally stem from community associations mostly composed of poorly educated residents. Residents of Pikine Sor Diagne also remain on the margins of the local council whose responsibilities encompass a larger area of Pikine. As a result, they struggle to capture the attention of international donors as well as their own local representatives.

These varying levels of participation and success in the quest for external investment produces unequal infrastructure in different neighbourhoods. These unequal dynamics in local development may in turn influence health inequalities.

### 3.2. Spatial Variations in Health Status and Statistical Associations

Given the distinct socio-territorial processes at work in the four studied neighbourhoods in Saint-Louis, we hypothesized that residents in these sites experience different levels of vulnerability to a variety of health conditions. In Table 3 we present our clinical findings for adults (age 35–59) in each of the four neighbourhood sites on being overweight, having high blood pressure, and being hyperglycaemic.

While we are cautious about arguing that socio-territorial processes have a direct causal effect on individual health status, our data reveal significant neighbourhood variation in the percentages of adults who were overweight and who registered high blood pressure and hyperglycaemia. Guet Ndar appears to have a significantly high percentage of adults who are overweight (77.1%), whereas the percentages of overweight adults in the other three neighbourhoods were all below 60%. The findings on high blood pressure demonstrate less variation. Nonetheless, there was a 14-point difference between the highest and lowest percentages of adults with high blood pressure: 42.5% in Guet Ndar vs. 28.5% in Léona. Our findings concerning adults with hyperglycaemia (a potential indication of type II diabetes) were the least variable by neighbourhood. Despite the fact that spatial variations are not statistically significant for hyperglycaemia, Guet Ndar had the highest percentage of adults whose fasting blood glucose levels were equal or higher than 110 mg/dL (12.6%), and Pikine Sor Diagne had the lowest percentage (6.8%).

At the scale of the full sample, these analyses reveal that age and sex are the factors most associated with high blood pressure, hyperglycaemia and overweight (see Table 4). We remain cautious about the importance of sex as a factor, since women represent 80% of our sample. Age was not statistically relevant in Guet Ndar or in Pikine for being overweight. Regardless of age, women’s diets seem to be quite similar in both places. However, for hyperglycaemia, age is associated with the rate of the disease only in Léona. Becoming older appears to be a risk factor of hyperglycaemia, specifically for residents of this area.

Sex had no effect on high blood pressure and hyperglycaemia at the scale of neighbourhoods (except for Pikine for hypertension), whereas it is very significant for overweight in every studied place. For the full sample, no education is associated with being overweight but not with hypertension and hyperglycaemia. This statistical linkage disappears when the hypothesis is tested at the neighbourhood scale.

Finally, socio-economic status does not influence the rates of three studied health conditions (hypertension, hyperglycaemia, and overweight), in any significant way to health status in the four neighbourhoods, except in Pikine Sor Diagne for hyperglycaemia (the more upper-class the household, the higher the rate hyperglycaemia). Certainly, disadvantaged populations are more affected by hypertension in Guet Ndar, but proportionally to the share of this socio-economic group in the area.

### 3.3. Health Inequalities and Care-Seeking Behaviour

We found few statistical associations between measured factors and rates of hypertension, hyperglycaemia, and being overweight. Despite this, disease rates vary significantly in the study neighbourhoods.

We have several hypotheses about how socio-territorial processes may contribute to this variation in morbidity by neighbourhood. At the individual level, being overweight, having high blood pressure, and having hyperglycaemia are reflections of diet, tobacco use, and levels of physical activity [28,29]. There was remarkable consensus about the etiology of hypertension and diabetes in our interviews with individuals who had been diagnosed with these conditions. In over two-thirds of our interviews, respondents mentioned the overconsumption of sugar, refined carbohydrates, animal fat, and oil, combined with a lack of physical activity, as the primary causes. The Senegalese diet, whose focal point is the noon meal of fried fish and fried white rice, also includes white bread, sugar-laden tea, and soft drinks. Nearly all respondents cited these items as the source of growing cases of hypertension and diabetes.

While the main staples of the urban Senegalese diet are fairly consistent across social groups irrespective of economic status, residents of Guet Ndar asserted that their diet is particularly rich in oil and fried fish. As such, Guet Ndar provides an illustrative example of how neighbourhood processes, particularly livelihood strategies and territorial identities, may shape individual diet and levels of physical activity. Our interviews demonstrate that residents of Guet Ndar report a particular affinity for overeating and for a diet rich in fat and oil that has become part of their collective identity. “The things we eat here in Guet Ndar, it is only us who eat them,” explained a 54-year-old diabetic woman. Another woman in her fifties who is also diabetic asserted, “If you are from Guet Ndar you love very oily *ceeb u gen* (fried fish and rice). It is our main dish. And if you are diabetic they forbid you to eat rice, and we love rice.” “In this neighbourhood we love sugar, oil, and too many spices, especially the (salty) bouillon we put in our meals” asserted a woman in her sixties with hypertension.

It was clear in our interviews that overconsumption is a particular mark of distinction for residents of Guet Ndar. This overconsumption is possible due in part to the fishing economy, arguably the most robust economic sector in Saint-Louis. Most households have reliable access to fresh fish for their own consumption and cash from the daily sale of fish. Additional territorial factors include the occupational trajectory of most Guet Ndarians, and the population density of the neighbourhood. Men who work as fishermen tend to retire from the intense physical labour on the fishing boats by age 50. Similarly, women engaged in domestic labour are relieved of their household tasks by their unmarried adult daughters or their daughters-in-law (who join their household upon marriage due to cultural norms and the shortage of available housing in the neighbourhood) by the time they are 50. These patterns lead to long periods of relative sedentariness starting in early middle age during which vulnerability to hypertension and diabetes may increase. Again, while we would not point to these factors as directly causing Guet Ndar’s high rates of overweight adults, and the higher percentages of adults with high blood pressure and hyperglycaemia, they seem to indicate a confluence of enabling socio-territorial dynamics. Although there are unique socio-territorial features in the other three neighbourhood sites, none of them seem to have produced health outcomes as distinctive as the patterns we found for Guet Ndar.

In addition to examining how socio-territorial processes may produce variable environmental exposures and disease vulnerability, our study aimed to determine if these processes shape the possibility of successfully managing hypertension and type II diabetes. We explored the diagnostic experiences and therapeutic itineraries of men and women living with hypertension and type II diabetes in each neighbourhood site. Our analysis highlights two key concepts: healthscapes and treatability.

Healthscapes builds upon the classic understanding of medical pluralism and aims to convey how people understand and navigate their therapeutic options. A healthscape is therefore, “an individual’s subjective vision of a landscape’s medical resources and institutions, limited by costs and accessibility …” [30]. This idea moves our analysis beyond the presence or absence of medical infrastructure to suggest that access to medical care and treatment decisions are embedded in socio-territorial processes. Importantly, the notion of healthscape gives equal analytic weight to the landscape of health resources and “the social experience of that landscape as it is viewed and experienced by different actors working and living within it” [30,31,32].

In our interviews, we posed questions about individuals’ experiences with diabetes and hypertension, their understanding of etiology, their initial medical consultations and first medical diagnosis of these conditions, the medical recommendations they had received, and their therapeutic itineraries and health-seeking practices. Although the number of interviews was modest (approximately 20 per neighbourhood), we sought to determine if there are shared healthscapes for individuals living in the same neighbourhood, and if there are distinctive healthscapes for people who are hypertensive or diabetic.

A number of important themes emerged in our interviews. In nearly all cases, individuals were diagnosed with hypertension or diabetes after the onset of life-disrupting symptoms (weight loss, frequent urination, headaches, dizziness, fatigue, blurred vision, partial paralysis). Fewer than ten of our respondents were diagnosed in the course of receiving routine medical screening or an annual medical exam. Once individuals began to experience symptoms, there were four factors that most frequently influenced their choice of location for an initial consultation: perception of urgency, proximity, cost, and referral by a trusted individual. Many individuals reported that the perceived seriousness of their symptoms (fainting, mild paralysis, severe vomiting, inability to speak) meant that they went to the Saint-Louis Hospital, a Level 1 medical facility that serves the region of Saint-Louis. For those who did not initiate care at the hospital directly, some reported that they went to the medical facility that was closest to them. In most cases, after they were screened for high blood pressure or diabetes, they were immediately referred to the specialists at the Saint -Louis Hospital for follow-up care. Others cited high prices and long wait times as reasons to avoid the Saint-Louis Hospital, so they chose facilities that were “within their means” and where the work ethic was “serious, where they treat you well and you don’t have to wait.” Some individuals reported that they made their initial choice of medical venue on the basis of a recommendation from someone in their social network.

While space does not allow for more detailed analysis here, we found that among our interview participants, healthscapes, or the perception of available and accessible medical options, do not have a significant territorial dimension. While proximity did matter in some instances, the perception of accessibility was not limited to options in the neighbourhood or even by the boundaries of the city itself. Even residents of Pikine, who we found had the fewest number of health facilities in close proximity, did not have healthscapes that were different from residents of other neighbourhoods. A small number of our interview participants were treated for acute episodes in the capital city Dakar, and some continue to seek regular care there. We found no identifiable pattern of care-seeking by neighbourhood. We suspect that because untreated hypertension and diabetes produce dramatic symptoms, individuals seek care at the limited number of facilities that are deemed appropriate for serious health conditions. For more routine care and for less serious problems (vaccinations, pre-natal care, diarrhoea, malaria), territorial factors such as proximity may weigh more heavily in patients’ therapeutic itineraries.

Our interviews about illness experiences in the four neighbourhoods also sought to understand potential variations in the treatability of diabetes and hypertension. As health research in sub-Saharan Africa increasingly addresses the emerging challenge of non-communicable diseases (NCDs), the idea of treatability conveys the presence or absence of health policies, funding, biomedical expertise and tools, and people’s knowledge of particular pathologies [33,34]. Whyte’s analysis of the treatability of hypertension and diabetes in Uganda points to a lack of public health resources and infrastructure, and she highlights the challenges facing patients who are proscribed pharmaceutical regimens and diet and lifestyle changes [34].

Saint-Louis offers insight into the treatability of hypertension and diabetes in a setting where biomedical infrastructure for monitoring NCDs and local knowledge of these pathologies is relatively robust. As a result, treatability of hypertension and diabetes reflects access to available medical care, the ability to purchase relatively expensive pharmaceuticals (most patients spend between US$20–$50 per month on prescription medications), and the tension between recommended dietary changes and deeply held notions of food culture and sociality. We identified a number of key themes in patient accounts of how they managed hypertension and diabetes. The most significant themes were: the expense of medications, the high cost of dietary recommendations, the difficulty of following dietary recommendations due to food preferences, and the reliance on family members, particularly siblings and children, to help pay medical costs.

Again, we sought to determine if there was a socio-territorial dimension to treatability, i.e., if diabetes and hypertension are more treatable in some neighbourhoods than for others. We discerned no patterns in the interview themes to suggest that treatability is linked to an individual’s place of residence in Saint-Louis. While a majority of our interview participants sought to comply with medical treatment, cost of prescriptions emerged as the main obstacle to treatability. Only six individuals in our sample had any form of medical insurance, and of those six two reported that their insurance provided them with only partial coverage. Therefore, the vast majority of people living with diabetes and hypertension must pay all of their costs out of pocket. In the best scenario, patients have uninterrupted access to pharmaceuticals and their treatment is continuous. At least half of our interview respondents cited the tremendous financial burden of purchasing medicines, and indicated that their treatment is sometimes episodic. Interviewees described running out of medications and needing several weeks to save enough money for refills, as well as delaying medical appointments and laboratory work because they had no money. A few other patients described reducing their prescribed doses to stretch out their medications, or taking their medications only when they experienced noticeable symptoms. For most of our respondents, the cost of medical treatment far exceeds their personal budgets, and they therefore receive regular financial assistance from another family member (typically a sibling or a child) for their medical care. Treatability of these conditions is therefore related not just to the presence of adequate medical infrastructure and the financial means of the patient, but also to the ability of extended families to mobilize the necessary financial resources to pay for treatment.

All of our interview participants described the dietary recommendations that are known to lower blood pressure and blood glucose levels: reduce sugar, salt, fatty foods, white rice, and other refined carbohydrates while increasing fresh fruits and vegetables, grilled fish and leaner meat. Interview participants described many obstacles to complying with their proscribed diet. Cost was a predominant concern in our interviews. Diabetes and hypertension were described as being diseases for the rich or bourgeois because of the combined expense of medications and diet. Healthy foods are said to be more expensive, and complying with dietary recommendations means that the household cook effectively has to prepare a separate meal three times a day. This additional domestic labour was simply not feasible in some households. As a result, some interviewees reported eating very small portions of the family’s meal at noon time and skipping the evening meal. The idea of an entire household changing its diet to accommodate the diabetic or hypertensive family member was absent in all of the interviews save one. One head of household in Ndioloffène with high blood pressure reported that he had imposed food restrictions on his household: less oil, less salt, and no bouillon. He was the only one of our interview respondents who had attempted to shift the entire household’s diet.

In addition to the high cost of preparing separate meals, interview participants described how “tiring” and “unappetizing” it is to eat meals that are low in salt, fat, and sugar. It would be hard to overstate the central place that *ceeb u jen* holds in Senegalese food culture, and some interviewees claimed that it was next to impossible to eliminate rice from their diet. Evening meals often include a meaty sauce eaten with pieces of white bread. The recommendation for those with high blood pressure to avoid salty foods and fatty meats was reported to be particularly difficult. One 80-year-old resident of Léona with hypertension explained his reaction to his physician’s recommendations, “He told me to give up meat, oil, and tea. I told him that he wanted me to die!” His incredulity at the idea of giving up food items that are central to the Senegalese diet illustrates the challenge of attempting to comply with dietary restrictions. An additional obstacle is that respecting dietary recommendations usually means eating alone while the rest of the family eats from a collective bowl. The challenge of eating in isolation appeared in 28 of our interviews.

In sum, treatability of hypertension and type II diabetes appears to be largely related to the cost of medications and the recommended diet. As such, treatability varies according to the financial means of the patient and his or her immediate household, and the social networks that can be mobilized to help pay for care. Further analysis might demonstrate more evidence that neighbourhood residence influences economic status and/or the density and social cohesion of one’s social networks. Nonetheless, the findings presented here do not support a clear socio-territorial influence on the treatability of hypertension and diabetes.

## 4. Discussion

Our study explored the social, environmental, and territorial processes that produce health disparities in urban West Africa. We suggest that these disparities, and vulnerability to disease more broadly, are the product of a constellation of forces that are the cumulative result of the social and political management of particular places over time. In particular, we sought to understand the complex relationship between socio-territorial processes and exposure to health risks, access to medical care, and health-seeking patterns. The data that we collected in Saint-Louis, Senegal demonstrates how the unique socio-spatial characteristics of different neighbourhoods both reflect their particular histories and play a role in shaping health.

### 4.1. Socio-Territorial Processes and Risk of Non-Communicable Disease

Our findings demonstrated distinct health patterns in the four neighbourhoods in terms of the numbers of individuals who were overweight and who showed signs of high blood pressure and hyperglycaemia. The largest difference was in the percentage of overweight individuals; our highest neighbourhood level was more than 20 points higher than the lowest level overweight individuals. For high blood pressure, we found a nearly 10-point difference, and for hyperglycaemia we found nearly a six-point difference. In Guet Ndar, 77.1% of the research participants were overweight, 42.5% had high blood pressure and 13.1% were hyperglycaemic at the time of the clinical exam. Ndioloffène was second to Guet Ndar in all three categories. These two neighbourhoods have some interesting similarities, such as the highest proportion of residents who were born in Saint-Louis, good access to water and electricity, and close proximity to a number of public medical facilities. While by our socio-economic proxies they seem to differ in terms of economic status, they are both wealthy neighbourhoods. As we explained previously, the proxies we used for class were not applicable in Guet Ndar. Rather than spend money on the household goods that we surveyed (televisions, refrigerators, etc.), we found that most wealth in Guet Ndar is instead invested in fishing equipment and consumption. (The cost of a wooden pirogue in 2010 was around US$6700 [26]). However, these neighbourhoods differ significantly in terms of level of schooling. In Guet Ndar, 69.5% of residents have never attended school, compared to 21.9% in Ndioloffène. In sum, the residents of Guet Ndar and Ndioloffène, the two wealthiest neighbourhoods in our study and two of the oldest in the city, appear to be the most vulnerable to being overweight and having hyperglycaemia and hypertension.

While wealth is assumed to ensure household food security, the ability to consume or even overconsume may be an important means of demonstrating class status and may serve to reinforce male household heads’ reputation as good providers [35,36]. The socio-cultural importance of being a good provider and of eating well likely may overshadow educational messages about healthy and balanced eating. The high proportion of residents in each neighbourhood who are native to Saint-Louis merits further investigation. The higher percentage of native-born residents may also indicate a level of homogeneity in social and cultural practices. In Guet Ndar, most men and women live a sedentary lifestyle by age 50. Whereas the fishing economy ensures early retirement in Guet Ndar, in Ndioloffène, it might be that higher levels of education are associated with employment that is sedentary in nature. Finally, it seems that close proximity to medical facilities in Guet Ndar and Ndioloffène is not a protective factor for being overweight or for having hypertension or diabetes. Proximity alone did not increase the likelihood of regular medical screenings, nor greater exposure to education campaigns that may increase prevention measures.

Overall, the neighbourhoods of Ndioloffène and Léona were the most similar in terms of being centrally located neighbourhoods that were the product of intentional urban planning in the colonial period. They have similar percentages of residents who were born in Saint-Louis, and are also quite comparable in terms of economic status and education. Given these similarities, we find it surprising that Ndioloffène’s population appears to be more at risk for hypertension. The greater proportion of Ndioloffène’s population that is upper class and the greater number that have attended school are probably factors which increase vulnerability. Middle-class populations are more represented in Léona, and this particular concentration could explain the lower prevalence of hypertension and hyperglycaemia.

Regarding this point particularly, it is interesting to focus on Pikine Sor Diagne. In Pikine we found that residents are equally distributed among our three socio-economic categories. Unlike Ndioloffène, Léona and Guet Ndar, which are quite fixed in their socio-spatial patterns, Pikine is presently undergoing a major socio-demographic transition due to land speculation. Upper-class and middle-class families are moving to the original fishing neighbourhood, which could be source of disruption to social cohesion. Given the growing heterogeneity of the neighbourhood, aggregate prevalence rates potentially mask income-related inequalities in residents’ health status. Most diagnosed cases of hyperglycaemia are however from upper-class populations. In the course of our research, we found that first impressions and readily visible features of the urban landscape are not necessarily indicative of the processes shaping health in a given neighbourhood. The assumption that wealthy neighbourhoods would be less healthy while poorer, unzoned neighbourhoods would be at less risk of hypertension and hyperglycaemia were challenged by our findings. Pikine, in particular, demonstrates that more complex socio-territorial processes are at work.

We also found tremendous variability in the ability of neighbourhood residents to mobilize collectively to attract development funding and to launch public health projects in the neighbourhood. Health education campaigns and other health promotion projects were visible in Ndioloffène and Léona. To our knowledge, there have been no donor-funded health education campaigns or grassroots health mobilizations in Pikine. The absence of this kind of community mobilization in Pikine could be explained by contemporary social mutations in the neighbourhood. Our focus on hypertension and hyperglycaemia did not allow us to draw any conclusions about levels of neighbourhood organizing and vulnerability to disease. Had we focused on malaria or another vector-borne illness, community efforts to reduce exposure by improving waste disposal, eliminating standing water, etc. might have had a more visible impact. While this neighbourhood capacity to mobilize collectively for local development may not have a direct effect on health status, it reveals important differences in social organization and in resident-led efforts to improve neighbourhood conditions.

### 4.2. Socio-Territorial Processes, Health Behaviour and Therapeutic Itineraries

Being overweight and developing high blood pressure and hyperglycaemia—the conditions that we focused on here—are most closely related to dietary practices and to levels of physical activity. On this point, the case of Pikine regarding hyperglycaemia is interesting: we hypothesize that because social patterns and cultural norms are currently changing at a fast pace in Pikine, socio-economic status was more significant than diet and other lifestyle factors (which were more important in other neighbourhoods. Our illness experience interviews asked diabetics and individuals suffering from hypertension about the etiology of these conditions, and in their own case, why they thought they had developed these conditions. In nearly every interview, respondents mentioned their own past dietary practices, with a particular focus on salt, oil, sugar, and fried fish and rice in the Senegalese diet. The consensus on etiology in our interviews was striking. Yet it was only in Guet Ndar that respondents expressed the idea that overeating and a particular love of oily rice was part of their socio-territorial identity as Guet Ndarians. The interviewees also asserted that regardless of economic status, residents of Guet Ndar have an extremely high level of food security, particularly access to fresh fish. Residents of Guet Ndar conveyed that overconsumption is a mark of distinction and a noteworthy trait among Guet Ndarians. As mentioned above, the high number of Guet Ndarians born in the city (and even in Guet Ndar itself), combined with high degrees of homogeneity and endogamy, have produced a strong collective identity, and particular social practices around food are central to this identity.

In addition to the ways that socio-territorial processes shape vulnerability to disease, we explored the extent to which these processes might shape health-seeking behaviour and therapeutic itineraries. We did not find any evidence to suggest that once individuals experience symptoms of hypertension or diabetes (or are diagnosed with these conditions as part of a medical exam) that neighbourhood influences access to medical care or therapeutic choices and medical strategies. We analysed our interviews by seeking themes related to both healthscapes (perceptions of accessible medical options) and treatability (the presence or absence of medical technology and knowledge, and the ability to make use of these tools for treatment), and found no discernible patterns at the neighbourhood level. It may be that at least for these non-communicable diseases, social and economic processes within the household are more influential than the socio-territorial processes at work at the neighbourhood level. In addition, because of a lack of preventative screening, most respondents were not diagnosed until well after the onset of disruptive symptoms. The near-universal perception that these symptoms were serious and that they required immediate medical intervention produced similar patterns of health seeking in which individuals went straight to the hospital or were referred immediately to the hospital by other providers. Again, we suspect that for other medical conditions, understandings of etiology and treatment and therapeutic itineraries may be more varied and may be more discernibly shaped by neighbourhood.

Nearly every interviewee had been prescribed a drug regimen and dietary restrictions as part of their initial diagnostic encounter. Our interviews demonstrated that patients in every neighbourhood faced significant challenges trying to comply with dietary recommendations. These challenges stem from cost, food preferences, and the household organization of labour which can limit a household’s ability to prepare separate meals. Patients in every neighbourhood also described the extremely high cost of follow-up medical appointments, lab work, and maintaining a supply of prescription drugs. Individuals in every neighbourhood had experienced ruptures in their treatment because of a lack of means to purchase recommended foods and drugs.

We see two potential underlying commonalities across our four neighbourhood sites that may help explain the lack of neighbourhood variation in health-seeking behaviour. First, there seems to be a widespread consensus about the etiology of hypertension and diabetes, and equally widespread familiarity with the biomedical recommendations for managing these diseases. While some respondents made use of traditional herbal medicines alongside their prescription drugs, fewer than five of our respondents questioned the efficacy of biomedicine for their condition. Given this powerful cultural consensus, treatability seems to reflect the purchasing power of the individual patient, or those in his or her social network who might help pay for food and medicine. We did find some inconsistencies in individuals’ understanding of the chronic nature of the condition. While most interviewees explained that there was no permanent cure for their problem, some suggested that they only needed to follow medical recommendations when they were experiencing symptoms. Nonetheless, patients must have access to medications that they can then decide to take or not take, and high costs were the primary barrier. Second, if economics is the most influential factor on treatment patterns, we might expect to see more variation based on relative wealth of the different neighbourhoods. But some patients in poor households are able to mobilize their social networks for assistance, and the medical regimen is so expensive that it poses an economic obstacle for even the wealthiest households. As such, we suspect that therapeutic itineraries are embedded in social and economic relations that must be understood at the scale of individuals and their social networks rather than at the neighbourhood level.

### 4.3. Limitations of the Study

There are several limitations to this study. The study respondents who agreed to receive a clinical exam and participate in the accompanying socio-medical questionnaire were overwhelmingly female. Our statistical analysis of the influence of sex on hypertension, hyperglycaemia, and being overweight might have produced a different outcome if there were an even number of males and females. We also had a difficult time identifying adequate socio-economic proxies that could capture the range of livelihood strategies and consumption patterns in Saint-Louis. While durable household goods and household infrastructure adequately reflect socio-economic status in some neighbourhoods, they were ineffective at conveying the relative wealth and employment security in Guet Ndar. More information on each neighbourhood at the outset of the study and more time devoted to preliminary field work on socio-spatial processes in each neighbourhood prior to the household survey may have helped to determine more effective proxies. Finally, we did not have the opportunity to conduct follow-up qualitative interviews with the individuals who showed signs of hypertension and hyperglycaemia during the clinical exams. We therefore cannot confirm if these individuals were ultimately diagnosed with hypertension or diabetes.

## 5. Conclusions

The SANTINELLES project explores the ways that socio-territorial processes produce urban places and can influence key dimensions of health: exposure to risks, health behaviour, accessibility of medical care, therapeutic itineraries, and health status. Our findings in Saint-Louis identified distinct socio-territorial processes at work in the four sampled neighbourhoods, revealing the contemporary effects of very different historical legacies, socio-economic realities, degrees of homogeneity, etc. While we do not suggest that socio-territorial processes exert a direct influence on individual bodies, the significant disparities we found between our four sites in terms of being overweight and having hypertension or hyperglycaemia appears to confirm the significance of analysis health disparities at this scale. Considering different scales of risk factors, specifically the neighbourhood scale, reveals the importance of territorial processes in the production of health inequalities. Significant variations exist in the study neighbourhoods between rates of overweight, and hypertension. Contributing factors like schooling level and socio-economic status are less correlated. These results support the argument for a socio-territorial approach to health: a constellation of processes influence health status at different scales. Both quantitative and qualitative research tools are required to capture complexity of places and local health inequalities.

Our findings also illustrate the potential for interdisciplinary, mixed-method approaches to urban health. While our geographic methods allowed us to identify and document a variety of socio-territorial dimensions of each neighbourhood, our anthropological approach to illness histories and illness narratives permitted us to engage with subjective experiences of health seeking and treatability in the midst of numerous economic and cultural constraints to optimum health. Our medical data on individuals from each neighbourhood increases our confidence that urban heterogeneity does in fact have implications for health status and health disparities. While our geographic methods documented great diversity in neighbourhood characteristics, our illness interviews demonstrated widespread consensus in understandings of hypertension and diabetes, and common challenges in living with these conditions. Our qualitative work on socio-territorial processes in each neighbourhood also allowed us to overcome the potential limitations of a purely quantitative approach. For example, the tremendous material wealth in Guet Ndar was not evident from socio-economic proxies in the household surveys and would have been missed without field research in the neighbourhood.

Finally, we found that some risk factors may exert tremendous influence in one neighbourhood and almost none in another. As such, we suggest that the collective influence of risk factors must be explored with the understanding that their relative importance may be mediated by other socio-territorial processes happening in a given locale [9]. The importance of any given risk factor cannot be assumed before it is examined within a particular socio-spatial context.

## Figures and Tables

**Table 1:** Characteristics of the socio-medical survey participants.

Variables		All Participants	Guet Ndar	Léona	Ndioloffène	Pikine sor Diagne	*p* Value
		815	214	186	224	191	
Age of participants	35–39	196 (24)	49 (22.9)	38 (20.4)	59 (26.3)	50 (26.2)	N.S
	40–44	181 (22.2)	40 (18.7)	45 (24.2)	46 (20.5)	50 (26.2)	
	45–49	133 (16.3)	37 (17.3)	27 (14.5)	35 (15.6)	34 (17.8)	
	50–54	155 (19)	41 (19.2)	41 (22)	44 (19.6)	29 (15.2)	
	≥55	146 (17.9)	47 (21.9)	32 (17.2)	40 (17.9)	27 (14.1)	
Sex of participants	Women	650 (79.8)	176 (82.2)	143 (76.9)	185 (82.6)	146 (76.4)	N.S
	Men	165 (21.2)	38 (17.8)	43 (23.1)	39 (17.4)	45 (23.6)	
Education level	Not educated	345 (42.3)	148 (69.2)	52 (28)	48 (21.4)	97 (50.8)	*p* < 0.01
Length of residence	Born in Saint-Louis	649 (79.6)	204 (95.3)	144 (77.4)	186 (83)	115 (60.2)	*p* < 0.05
Neighbourhood satisfaction	Wish to stay in their neighbourhood	394 (48.3)	35 (16.4)	91 (48.9)	128 (57.1)	140 (73.3)	*p* < 0.01
Perception of health status	Poor	191 (23.4)	42 (19.6)	42 (22.6)	37 (16.5)	70 (36.6)	*p* < 0.01
	Correct	270 (33.1)	77 (36)	43 (23.1)	92 (41.1)	58 (30.4)	
	Good to excellent	348 (42.7)	95 (44.4)	98 (52.7)	92 (41.1)	63 (33)	

Percentages between parentheses; N.S: Not significant.

**Table 2:** Categorization of the four studied neighbourhoods by socio-territorial organization and health status.

**Variables**			**Scale of Variables**	**Guet Ndar**	**Pikine**	**Ndioloffène**	**Léona**
**Socio-spatial context**	**Age of the neighbourhood**		Neighbourhood level	Precolonial district: traditional Wolof fishing neighbourhood	Precolonial village of fishermen and farmers: late integration to Saint-Louis as an administrative district (1980s)	Created during the colonial period (beginning of the 19th century): socially and ethnically mixed neighbourhood with some gentrification	Created during the colonial period (mid-20th century): middle to upper class households, historically served as colonial civil servants
	**Length of residence in Saint-Louis (percentage of people born in the city)**		Individual level	96.20%	60.50%	83.00%	78.70%
	**Spatial Heterogeneity**	**Built density**	Neighbourhood level	Very high	Low	Low	Medium
		**Physical proximity to health care structures**		Very good	Poor	Very poor	Good
		**Availability of water and electricity**		Household access to water and electricity	Ongoing plans to improve for municipal water and electric supply systems	Household access to water and electricity	Household access to water and electricity
	**Economic status**	**Poor**	Household level	58.50%	34.20%	20.30%	19.50%
		**Middle class**		31.30%	33.70%	28.90%	38.60%
		**Upper class**		10.20%	32.10%	50.80%	41.90%
	**Education level**	**Not educated**	Individual level	69.20%	50.80%	21.40%	28.00%
**Socio-territorial organization**	**Perceptions of the neighbourhood (verbatim records from interviews)**		Neighbourhood level	“There is a boundary between Saint-Louis and Guet Ndar.”	“They are sectarian people. It is a neighbourhood of fishermen: they are very close.”	“The neighbourhood is one the hubs of Saint-Louis’ intelligentsia.”	“It is a neighbourhood of intellectuals. […] in Léona, we are urban dwellers, with proper urban practices.”
	**Organization of political actors and network for local development**		Neighbourhood level	Cooperation between the local council and the municipal government which directs external funds to Guet Ndar. But lack of legitimacy of the local council among residents who use their own networks to finance neighbourhood projects.	No representation of Sor Diagne residents in the local council. Modest and sporadic projects of local development led by community associations.	Omnipresence of powerbrokers who govern the local council according to their own political ends. They secure significant amounts of external and internal funding for major projects.	Omnipresence of the local council which coordinates local actors for various large projects. Increasing autonomy from the municipal government thanks to tremendous fundraising capacity.
	**Presence of health promotion and prevention projects**		Neighbourhood level	Several days of free medical consultations have been organized, and an ambulance was purchased by external donors through a key powerbroker.	A small dispensary was built with external financing. Free medical consultations have been organized for non-communicable diseases screening, implemented by the greater Pikine council at the Sor Daga health post.	Several days of free medical consultations and follow-up for chronic disease patients have been organized and financed by local powerbrokers.	Several campaigns for health prevention and follow-up for chronic disease patients were funded by international donors. Renovation of the local health post was paid for by external funding.
**Variables**			**Scale of Variables**	**Guet Ndar**	**Pikine**	**Ndioloffène**	**Léona**
**Health status**	**Overweight**		Neighbourhood level	77.10%	56.50%	59.80%	56.50%
	**Prevalence of high blood pressure**			42.50%	32.50%	38.40%	28.50%
	**Prevalence of hyperglycaemia**			12.60%	6.80%	8.90%	7.50%

**Table 3:** Health status of socio-medical survey participants.

Health Indicators	Full Sample	Guet Ndar	Léona	Ndioloffène	Pikine Sor Diagne	*p* Value
	815	214	186	224	191	
Overweight	512 (62.8)	165 (77.1)	105 (56.5)	134 (59.8)	108 (56.5)	*p* < 0.01
High blood pressure	292 (35.8)	91 (42.5)	53 (28.5)	86 (38.4)	62 (32.5)	*p* < 0.05
Hyperglycaemia	74 (9.1)	27 (12.6)	14 (7.5)	20 (8.9)	13 (6.8)	N.S

Percentages between parentheses; N.S: Not significant.

**Table 4:** Statistical associations between contributing factors and health status.

**Overweight (A = Affected/NA = Non-Affected)**																
**Contributing Factor**		**Total (%)**			**Guet Ndar (%)**			**Léona (%)**			**Ndioloffène (%)**			**Pikine Sor Diagne (%)**		
		**A**	**NA**	***p* Value**	**A**	**NA**	***p* Value**	**A**	**NA**	***p* Value**	**A**	**NA**	***p* Value**	**A**	**NA**	***p* Value**
Sex	Female	56.9	22.8	*p* < 0.01	69.2	13.1	*p* < 0.01	52.7	24.2	*p* < 0.01	54.9	27.7	*p* < 0.01	49.7	26.7	*p* < 0.01
	Male	5.8	14.0		7.9	9.8		3.2	18.3		4.9	12.5		6.8	16.2	
Age	35–39	11.8	12.3	*p* < 0.01	15.9	7.0	N.S	7.0	13.4	*p* < 0.01	10.7	15.6	*p* < 0.01	13.1	13.1	N.S
	40–44	14.2	8.0		15.0	3.7		14.5	9.7		13.8	6.7		13.6	12.6	
	45–49	10.2	6.1		13.6	3.7		8.6	5.9		8.9	6.7		9.4	8.4	
	50–54	12.8	6.3		13.6	5.6		11.3	10.8		14.3	5.4		11.5	3.7	
	≥55	13.6	4.3		19.2	2.8		14.0	3.2		12.1	5.8		8.9	5.2	
Education level	Educated	32.3	23.1	*p* < 0.05	21.0	9.3	N.S	34.4	29.0	N.S	46.9	31.7	N.S	25.7	22.5	N.S
	Not educated	29.4	12.9		55.6	13.6		18.3	9.7		12.9	8.5		30.4	20.4	
Economic status of household	Poor	21.3	11.2	N.S	44.9	12.1	N.S	10.8	7.5	N.S	9.8	10.7	N.S	18.8	14.1	N.S
	Middle class	21.1	12.0		23.8	7.9		23.1	14.0		19.6	11.2		17.8	15.7	
	Upper class	19.5	13.6		6.5	2.8		21.0	21.0		30.4	18.3		19.9	13.1	
**High Blood Pressure (A = Affected/NA = Non-Affected)**																
**Contributing Factor**		**Total (%)**			**Guet Ndar (%)**			**Léona (%)**			**Ndioloffène (%)**			**Pikine Sor Diagne (%)**		
		**A**	**NA**	***p* Value**	**A**	**NA**	***p* Value**	**A**	**NA**	***p* Value**	**A**	**NA**	***p* Value**	**A**	**NA**	***p* Value**
Sex	Female	30.9	48.8	*p* < 0.01	36.9	45.3	N.S	23.7	53.2	N.S	32.6	50.0	N.S	29.3	47.1	*p* < 0.01
	Male	4.9	14.8		5.6	12.1		4.8	16.7		5.8	11.6		3.1	19.9	
Age	35–39	4.5	19.5	*p* < 0.01	6.5	16.4	*p* < 0.01	2.7	17.7	*p* < 0.05	4.9	21.4	*p* < 0.01	3.7	22.5	*p* < 0.01
	40–44	6.7	15.5		7.0	11.7		5.4	18.8		8.0	12.5		19.9	6.3	
	45–49	4.7	11.7		5.1	12.1		4.8	9.7		2.7	12.9		6.3	11.5	
	50–54	8.8	10.2		10.3	8.9		5.9	16.1		10.7	8.9		7.9	7.3	
	≥55	10.8	7.1		13.6	8.4		8.6	8.6		12.1	5.8		8.4	5.8	
Education level	Educated	19.6	35.7	N.S	12.6	17.8	N.S	18.8	44.6	N.S	30.8	47.8	N.S	15.2	33.0	N.S
	Not educated	16.0	26.4		29.9	39.3		8.6	19.4		7.6	13.8		17.3	33.5	
Economic status of household	Poor	12.5	20.0	N.S	26.2	30.8	N.S	3.8	14.5	N.S	8.0	12.5	N.S	11.0	22.0	N.S
	Middle class	11.5	21.6		10.3	21.5		12.4	24.7		13.8	17.0		9.4	24.1	
	Upper class	11.3	21.8		5.1	4.2		11.3	30.6		16.5	32.1		12.0	20.9	
**Hyperglycaemia (A = Affected/NA = Non-Affected)**																
**Contributing Factor**		**Total (%)**			**Guet Ndar (%)**			**Léona (%)**			**Ndioloffène (%)**			**Pikine Sor Diagne (%)**		
		**A**	**NA**	***p* Value**	**A**	**NA**	***p* Value**	**A**	**NA**	***p* Value**	**A**	**NA**	***p* Value**	**A**	**NA**	***p* Value**
Sex	Woman	8.0	71.8	*p* < 0.05	11.2	71.0	N.S	6.5	70.4	N.S	8.5	74.1	N.S	5.2	71.2	N.S
	Man	1.0	19.0		1.4	16.4		0.5	21.5		0.4	17.0		1.6	22.0	
Age	35–39	1.2	22.8	*p* < 0.01	2.3	20.6	N.S	0.5	19.9	*p* < 0.01	1.3	25.0	N.S	0.5	25.7	N.S
	40–44	1.5	20.7		1.4	17.3		1.1	23.1		1.3	19.2		2.1	24.1	
	45–49	1.8	14.5		3.3	14.0		1.1	13.4		1.3	14.3		1.6	16.2	
	50–54	1.7	17.3		2.8	16.4		1.1	21.0		1.8	17.9		1.0	14.1	
	≥55	2.5	15.5		2.8	19.2		2.7	14.5		3.1	14.7		1.0	13.1	
Education level	Educated	4.8	50.8	N.S	2.3	28.0	N.S	4.8	59.1	N.S	6.7	71.9	N.S	5.2	43.5	N.S
	Not educated	4.0	38.3		10.3	58.9		1.6	26.3		2.2	19.2		1.6	49.2	
Economic status of household	Poor	2.9	29.6	N.S	8.4	48.6	N.S	0.0	18.3	N.S	2.2	18.3	N.S	0.5	32.5	*p* < 0.05
	Middle class	2.3	30.9		1.9	29.9		4.8	32.3		1.3	29.5		1.6	32.5	
	Upper class	3.7	29.6		1.9	7.5		2.7	39.8		5.4	43.3		4.7	28.3	
