# Correction: Vialard, L., et al. Toward a Socio-Territorial Approach to Health: Health Equity in West Africa. *Int. J. Environ. Res. Public Health* 2017, *14*, 106

**DOI:** 10.3390/ijerph14060608

**Published:** 2017-06-08

**Authors:** Lucie Vialard, Clara Squiban, Gilles Riveau, Emmanuel Hermann, Doudou Diop, Florence Fournet, Gérard Salem, Ellen E. Foley

**Affiliations:** 1 Laboratoire Dynamiques Sociales et Recomposition des Espaces (LADYSS), Université Paris Ouest Nanterre, 92000 Nanterre, France; lucie.vialard@gmail.com (L.V.); clara.squiban@gmail.com (C.S.); salem.gerard@gmail.com (G.S.); 2 Biomedical Research Center EPLS, BP 226, Saint-Louis, Senegal; gilles.riveau@gmail.com (G.R.); doudou.diop@espoir-sante.org (D.D.); 3 Institut Pasteur of Lille, University of Lille, CNRS, Inserm, CHU Lille, Institut Pasteur de Lille, U1019-UMR 8204-CIIL-Center for Infection and Immunity of Lille, F-59000 Lille, France; emmanuel.hermann@univ-lille2.fr; 4 Unité Mixte de Recherche Maladies Infectieuses et Vecteurs: Ecologie, Génétique, Evolution et Contrôle (MIVEGEC), Institut de Recherche pour le Développement, 34394 Montpellier, France; florence.fournet@ird.fr; 5 Centre Population et Développement (CEPED), Université Paris Descartes-Institut de Recherche pour le Développement, 75006 Paris, France; 6 International Development and Social Change, IDCE, Clark University, Worcester, MA 01610, USA

In the original version of our article [1], insufficient acknowledgement was given to the organizers of the medical survey. We apologize for this mistake. To correct this oversight, Gilles Riveau, Doudou Diop and Hermann Emmanuel have been added as authors.

The corrected list of authors and author contributions are provided below:

Lucie Vialard, Clara Squiban, Gilles Riveau, Emmanuel Hermann, Doudou Diop, Florence Fournet, Gérard Salem and Ellen E. Foley

The manuscript will be updated and the original will remain online on the article webpage, with a reference to this Correction.

## References

[1] Vialard L., Squiban C., Fournet F., Salem G., Foley E.E. (2017). Toward a socio-territorial approach to health: Health equity in West Africa. Int. J. Environ. Res. Public Health.

